# An Update on GLP‐1 Receptor Agonists

**DOI:** 10.1111/1753-0407.70147

**Published:** 2025-08-22

**Authors:** Zachary Bloomgarden

**Affiliations:** ^1^ Department of Medicine, Division of Endocrinology, Diabetes and Bone Disease Icahn School of Medicine at Mount Sinai New York New York USA

The glucagon‐like peptide‐1 (GLP‐1) receptor agonists (GLP‐1RAs) are a class of medications primarily developed for the management of type 2 diabetes (T2D) and are increasingly being used for various other health effects. Here's a simplified overview of their history, effects, and future prospects:

## 1 History and Development

Glucose‐dependent insulinotropic polypeptide (GIP) and then GLP‐1 were identified in the 1970s as peptides potentiating the insulin secretory response to nutrient ingestion. In the early 1980s, the proglucagon amino acid sequence and gene were mapped, leading to the recognition of the common derivation of GLP‐1 and GLP‐2, produced in the L‐cells of the distal small intestine, while GIP is produced in the K‐cells of the proximal small intestine. The first therapeutically used GLP‐1RA was isolated as exendin‐4 from the saliva of a venomous lizard, *Heloderma suspectum* (the “Gila monster”), in 1992, with initial preclinical studies reported in 1996; although FDA approval for its use in treatment of T2D as exenatide was not granted until 2005. The next GLP‐1RA used therapeutically was liraglutide, receiving approval in 2010, with dulaglutide receiving approval in 2014, semaglutide approval in 2017, and tirzepatide (a combined agonist of GLP‐1 and GIP) approval in 2022; FDA approvals of liraglutide, semaglutide, and tirzepatide for treatment of obesity were granted in 2014, 2021, and 2023, respectively.

## 2 Therapeutic Effects in Diabetes: Metabolic Benefit

Both the GLP‐1 and GIP receptor agonists enhance insulin production when glucose levels are elevated. This physiological insulin‐secretory effect contrasts with the action of the older sulfonylureas, which activate the ATP‐sensitive potassium channels of the pancreatic beta cells, mimicking the cellular process that physiologically acts to link insulin production to the beta cell's energy state. The consequent increase in endogenous insulin production leads the GLP‐1RAs to have glucose‐lowering action comparable to that of daily treatment with basal insulin [1]; with weight loss rather than weight gain and with lower likelihood of hypoglycemia [2, 3].

## 3 Therapeutic Effects in Diabetes: Cardiovascular (CV) Outcome Benefit

In addition to their glycemic benefits, GLP‐1RAs are associated with improvement in mortality and in CV and renal outcomes in clinical trials [4, 5], both in people having and not having T2D [6], as well as in real‐world studies of people with T2D [7] and of people with obesity without T2D [8]. In a study from the US Veteran's Affairs hospitals with mean followup of nearly 4 years comparing more than 200 000 persons receiving GLP‐1RA with more than 1.2 million receiving usual care, benefits of GLP‐1RAs included reduction in risk of stroke, myocardial infarction, pulmonary embolism, phlebitis, heart failure, hepatic failure, chronic kidney disease, bacterial infections, postprocedural respiratory complications, aspiration pneumonitis, chronic obstructive pulmonary disease, pneumonia and respiratory failure, as well as improvement in a variety of psychiatric issues including suicidal ideation and cannabis, alcohol and opioid use disorders [9].

There is, however, heterogeneity in the response to GLP‐1RA in real‐world studies of people with T2D, with a study of more than 4000 people with T2D showing that 43% had neither weight loss nor glycemic improvement, and that the degree of weight loss was mild, at 1.4% of basal, with a mean HbA1c decrease of just 0.49% [10]. Furthermore, in a Danish study of over 40 000 first‐time users of GLP‐1RAs with T2D followed between 2007 and 2020, 14.1% and 21.2% discontinued at 6 and 12 months, respectively [11].

A crucial question has been whether the combined GLP‐1 and GIP receptor agonist tirzepatide is also associated with CV outcome benefit. On July 31, 2025, Eli Lilly and Co announced preliminary results of a nearly 5‐year head‐to‐head randomized controlled CV outcome trial, SURPASS‐CVOT, comparing tirzepatide with the GLP‐1RA dulaglutide among 13 299 high‐risk people with T2D [12]. The rationale for the use of an active comparator was the recognized benefit of GLP‐1RA in such patients, with the use of a placebo considered unethical. The combined risk of Major Adverse Cardiovascular Events (MACE) comprised of CV mortality, myocardial infarction, or stroke, was 8% lower for tirzepatide than for dulaglutide (numerically, albeit not statistically significantly, better), satisfying the criteria for non‐inferiority, with a significant reduction when combined with patient‐level data from the earlier trial of dulaglutide vs. placebo [13]. There was a significant reduction in secondary endpoints, although not correcting for multiple comparisons: 16% lower all‐cause mortality, slower rate of decline in the estimated glomerular filtration rate, greater reduction in HbA1c (1.7% vs. 0.9% from baseline of 8.4%) and 12% vs. 5% weight loss from a baseline of 93 kg. GI side effects occurred more frequently with tirzepatide than dulaglutide, with 13% vs. 10% of participants discontinuing treatment due to adverse events (12).

## 4 Combination Therapies

There is growing interest in combining GLP‐1RAs with other diabetes medications, particularly SGLT2 inhibitors. This combination appears to enhance cardiovascular and renal health, showing lower risks of heart failure and mortality than among individuals using GLP‐1RA alone [14].

## 5 Outcomes Beyond MACE

Emerging research suggests GLP‐1RAs may protect against neurodegenerative diseases, including Alzheimer's Disease [15] and Parkinson's Disease [16], with beneficial impacts on cognitive function [17]; although some of this may simply reflect GLP‐1RA‐related reduction in stroke. Metabolic‐associated steatotic liver disease affects some two‐thirds of people worldwide with T2D, of whom two‐thirds have steatohepatitis, with multiple studies showing evidence of benefit of GLP‐1RA with and without diabetes [18, 19]. GLP‐1RAs are associated with a reduction in the apnea‐hypopnea index among people with obstructive sleep apnea, with tirzepatide appearing to be more potent in this regard [20]. Innovation in GLP‐1RA formulations includes oral options and dual‐ and triple‐acting drugs targeting GIP, glucagon, and amylin as well as GLP‐1 for glucose and weight management. New agents and biosimilars are being tested. Orally active GLP‐1RAs have similar, although less potent, metabolic, weight‐reducing, and CV‐protective effects to those seen with parenterally administered GLP‐1RAs.

Akin to the “statin hypothesis” that statins, to a degree proportional to their cholesterol‐lowering effect, can prevent or treat CV disease, we appear to be close to confirming a “GLP‐1RA hypothesis” that this extended class of agents can prevent or treat CV disease, to a degree proportional to their weight‐ and glucose‐lowering effect, with multiple additional benefits. However, our very success with these agents has led to a dilemma: nearly 200 million people worldwide have T2D and high CVD risk [21]. How can we ensure that these effective but highly expensive medications can be afforded by all the patients we now recognize should be so treated?

## Conflicts of Interest

The author declares no conflicts of interest.
